# The Effects of Transglutaminase and Refrigerated Storage on the Physicochemical Properties of Whole Wheat Dough and Noodles

**DOI:** 10.3390/foods10071675

**Published:** 2021-07-20

**Authors:** Min Jeong Kang, Seo-Jin Chung, Sang Sook Kim

**Affiliations:** 1 Division of Strategic Food Technology Research, Korea Food Research Institute, Wanju-gun 55365, Korea; kmjkdy@gmail.com; 2 Department of Food and Nutrition, Ewha Womans University, Seoul 03760, Korea; sc79d@ewha.ac.kr

**Keywords:** whole wheat dough, transglutaminase, refrigerated storage, water extractable arabinoxylan, noodles

## Abstract

This study investigated the effects of transglutaminase (TG) concentrations (0, 0.1% and 1%) on the physicochemical properties of whole wheat dough (WWD) and noodles (WWN) during refrigerated storage (0, 1, 2, and 3 days). The yield, ferulic acid (FA) content, molecular weight (Mw), and apparent viscosity (AV) of water extractable arabinoxylan (WEAX) from refrigerated WWDs were analysed. The WEAX yield and FA tended to increase with refrigerated storage, while the Mw decreased. WEAX FA of from WWD with TG tended to be smaller than the control during refrigeration. The AV for all WEAXs gradually decreased during refrigeration. The TG concentration effects on WWD resistance to extension and extensibility and the WWN cooking properties and texture profile analysis (TPA) were studied. The water absorption and swelling index tended to decrease in WWNs with TG depending on refrigeration time compared to the control samples. The TPA results showed that WWNs with TG were significantly harder than the control after two days of refrigeration. This study demonstrated that TG affected not only WWD composition but also WWN physical properties during refrigerated storage.

## 1. Introduction

Wheat (*Triticum aestivum* L.) is one of the major food sources consumed globally. Whole wheat grain includes endosperm, germ, and bran. Bran is removed when refining flour, while whole wheat grain provides many dietary fibres and phytochemicals [1]. In addition, it is associated with health benefits that prevent chronic diseases such as obesity, diabetes, and heart diseases [2,3]. Across the world, people are interested in healthy foods that are conducive to preventing chronic diseases. Consumption of meals utilizing whole grain wheat has increased with the health consciousness of consumers. However, the bran fraction induces a conformational change in the gluten structure, changing from a viscoelastic β-spiral to an intermolecular β-sheet structure [4]. In other words, bran dehydrates the gluten network by its water absorption capacity and reduces the dough viscoelastic properties [4]. Furthermore, the bran addition induces rough mouthfeel, which causes poor whole wheat product quality. As a result, many research scientists have attempted to improve whole wheat product quality.

To improve the quality of whole wheat products, numerous studies have utilized various additives to induce structural changes in diverse whole wheat products [5,6,7,8]. Van et al. [5] reported that cellulase or pentosanase improved the whole wheat waxy bread quality. Niu et al. [9] explained that phosphate addition develops cooked noodle textures. Among the enzymes, transglutaminase (TG) can ameliorate a weak gluten network and transform it into a strong network [10]. TG is a transferase enzyme that constructs an isopeptide bond between glutamine and lysine amino acids [11]. Since gluten is composed of glutenin and gliadin, which have relatively higher lysine and glutamine contents, respectively [12], TG could provide a cross-linked structure within gluten. Niu et al. [7] found that TG makes a compact gluten network from whole wheat dough (WWD), confirming that the springiness and resilience of whole wheat noodles (WWN) increased. Consequently, TGs have been used to improve WWN quality [13,14].

Refrigerated product storage between 4 °C and 7 °C, is a convenient and cost-saving method for preparing flour products in advance [15]. The refrigerated dough industry also accounts for one of the fastest-growing parts of the ready-to-use grain-based industry [16]. Because the refrigerated dough market includes a wide range of products, WWD applicability could be expected to widen. However, during refrigerated storage, water migration or flour native enzymes, such as endoxylanase, change diverse dough components, modifying the water holding capacity [16].

One of the main components that modifies the water availability of refrigerated dough is arabinoxylan [17]. Arabinoxylan (AX) is a major non-starch polysaccharide in wheat that has a strong water holding capacity. AX is composed of β-1,4-linked D-xylopyranosyl residues as a backbone that are substituted with monomeric α-L-arabinofuranose units [18]. Some arabinose residues are esterified with ferulic acid (FA) at the O-5 position [19]. Among them, water extractable arabinoxylan (WEAX) especially affects starch water availability [20], and it influences dough viscosity [21]. Since WEAX has hydrophilic characteristics, it provided more significant physicochemical functions than water unextractable arabinoxylan [22]. In addition, WEAX FA interacts with the gluten network, providing a reaction between the free gluten protein SH and the WEAX-gluten mixed system [23].

One of the problems in refrigerated refined flour dough is the loss of dough strength [17]. Various additives or enzymes are applied to enhance the refrigerated dough product quality. For example, Tao et al. [24] used several additives, and after 5 days of storage found decreased and increased bread hardness caused by xylanase and hydrocolloids, respectively. Additionally, Whitney et al. [17] utilized glucose oxidase (GOX) for refrigerated dough development and explained that GOX could maintain the dough rheological properties. The enzymes in refrigerated dough are likely to modify the native action, namely component breakdown and refrigerated dough property maintenance or improvement.

Noodles are one of the major foods in Asia and are familiar to the rest of the world. Most studies on WWN quality have focused on dough rheological properties [25,26,27]. As mentioned above, numerous studies have reported WWN quality with various additives such as phosphate [9], and TG as well as emulsifiers [7]. The addition of inorganic phosphates enhanced WWN quality by decreasing the hardness of cooked noodles and increasing the springiness, cohesiveness and resilience [9]. TG and SSL were suggested as effective ingredients in enhancing the gluten strength of WWD and improving the qualities of WWN [7]. TG improved WWD stability by forming compact gluten structure and sodium stearoyl lactate (SSL) enhanced gluten strength. TG and SSL improved sensory properties of noodles such as bite, springiness, and mouth-feel [7].

However, information on WWDs or WWNs with TGs during refrigerated storage is scarce. Transglutaminase (TG) is known to form isopeptide bonds within the gluten network. Since one of the problems in refrigerated refined flour dough is the loss of dough strength [17], information on TG effects on retarding WWD strength loss is needed. Therefore, the purpose of this study was to investigate the TG effect on refrigerated WWD and WWN as well as on WEAX properties. Moreover, the correlation between WWN qualities and its components, WEAX and gluten, was studied.

## 2. Materials and Methods

### 2.1. Materials

The wheat samples used in this study were the Keumkang cultivar (Yeonggwang, Korea; geographic coordinates: 35°16′ N, 126°30′ E), which is a typical medium wheat in Korea. Whole wheat flour was obtained by grinding with an Ultra Fine Particle Mill DSCH 550S (Duksan Co., Ltd., Siheung, Korea). The moisture content and mean (d50) particle size of the WWF were 6.17% and 44.78 μm, respectively. TG (ACTIVA TG-B) was purchased from Ajinomoto (Tokyo, Japan). Alpha-amylase (Termamyl 120 L) and amyloglucosidase (AMG 300 L) were purchased from Novozymes (Bagsvaerd, Denmark). All reagents were analytical grade.

### 2.2. Whole Wheat Dough (WWD) and Noodle (WWN) Preparation

The dough preparation procedure followed the AACC method 66-60.01 [28]. Enzymes and salt were dissolved in the water before adding water to the flour. The dough formulation for a 400 g batch was as follows: flour—100%, sodium chloride (NaCl)—1% *w/w* flour weight basis (fwb), distilled water—37.5% (based on 14% flour moisture content), and TG—0.1%/1% *w/w* (fwb). The TG concentrations used in this study were determined based on the minimum and maximum concentrations for foods recommended by the supplier. The solution (water + enzyme + NaCl) was mixed for 1 min at 60 rpm using a mixer (K5SS, KitchenAid, Benton Harbour, MI, USA). Then, flour was added to the bowl in solution by mixing it at 135 rpm for 3 min. The resulting dough was rounded and wrapped in plastic film. WWD samples were used for WWN preparation initially and after 1, 2, and 3 days of storage at 3 + 1 °C.

WWN samples were made by method AACC 66-60.01 [28] with some modifications to produce whole wheat noodles properly since method AACC 66-60.01 is based on refined wheat flour dough. At each storage time, the dough was unfolded with a rolling pin and transferred to a dough sheeter (National Manufacturing Co. Inc., Chatham, MA, USA) with a 3 mm gap to make a sheet. The resulting sheet was folded in two and passed through the same gap again. The sheet that passed the roller gap was triple folded (from both ends to the middle) and passed through again. The final sheet was placed into a plastic bag and allowed to rest in an incubator (FP-201, Daeyung Bakery Machinery Co. Ltd., Seoul, Korea) for 60 min at 25 °C with 65% relative humidity. After resting, the sheet was passed through the roller with gaps of 2.4 mm and then 2.2 mm, which was also used for determining the dough dynamic rheological properties. After passing the roller two times, the sheet was cut into noodle strands (170 mm long and 4 mm wide) for measuring Kieffer extensibility, and then was used for cooking and texture profile analysis.

### 2.3. Extraction of Water Extractable Arabinoxylans (WEAX) from WWD

The water extractable arabinoxylan (WEAX) was extracted according to the methods described by Mansberger et al. [29] with some modifications. Briefly, freeze-dried dough ground with mortar was heated at 130 °C for 90 min in a dry oven to inactivate the enzymes. After cooling the materials, 50 g of sample was mixed with 500 mL deionized water and 0.01 mL α-amylase and stirred at 65 °C for 90 min in a water bath. Then, solids were eliminated after centrifugation at 5000× *g* for 10 min. The supernatant pH was set to 6.5, and 0.3 mL amyloglucosidase was added. Then, the supernatant was kept at 55 °C for 16 h for degradation of liquefied starch by amyloglucosidase. After the reaction ended, the enzymes were inactivated by boiling in a water bath for 30 min. After passing the resulting solution through filter paper (No 29, Hyundai Micro, Seoul, Korea), 10% (*v/v*) bentonite solution (0.2% mass) was added to remove proteins and stirred for 30 min at room temperature (RT). The pellet was removed again by centrifugation (5000× *g*, 10 min).

To isolate arabinoxylan from the supernatant, ethanol (100%, Sigma Aldrich, St. Louis, MO, USA) was added until the solution alcohol concentration reached 65% (*v/v*), and the solution was stirred for 30 min at RT. The isolated pellet was washed with 200 mL of 65% (*v/v*) ethanol and twice with 50 mL of acetone. The final material was air-dried for 16 h. The dried pellet was resolved in 200 mL of deionized water, and the supernatant induced by centrifugation was freeze dried.

### 2.4. Physicochemical Properties of WEAX from WWDs

#### 2.4.1. Molecular Weight of WEAX

The molecular weight (Mw) of WEAX was determined with a Tosoh EcoSEC HLC-8320GPC (Tosoh, Tokyo, Japan) using EcoSEC software. WEAX sample aliquots were solubilized in 0.1 M NaNO_3_ and filtered with a 0.45 μm nylon filter. The solutions (200 μL) obtained were separated on TSKgel GMPWxl and TSKgel G2500PWxl (7.8 mm ID × 300 mm, Tosoh, Tokyo, Japan) columns by isocratic elution with 0.1 M NaNO_3_ monitored with an RI detector. The flow rate was 1 mL/min, and the temperature was set at 40 °C. Pullulans (P50 to P800) were used for estimating Mw as a standard calibration.

#### 2.4.2. The Apparent Viscosity (AV) of WEAX Solutions

The apparent viscosity (AV) of WEAX aqueous solution (4%, *w/v*) was measured using an MCR 102 rheometer (Anton Paar, Graz, Austria) at 25 °C according to Simsek et al. [21]. After the WEAX was completely dissolved in deionized water, the solution was placed between two parallel plates (50 mm diameter) with a 500 μm gap. Flow curve tests were performed to measure viscosity as a function of the shear rate from 1 to 1200 s^−1^.

#### 2.4.3. Ferulic Acid (FA) Content of WEAX

The content of ferulic acid (FA) in WEAX was measured with a modified method by Wang et al. [22]. WEAX (50 mg) was dissolved in 3 mL of 4 M sodium hydroxide and saponified under a nitrogen atmosphere for 18 h at RT with continuous mixing on a Bio RS-24 Mini-Rotator (SIA Biosan, Riga, Latvia). Then, the supernatant was separated by centrifugation (3000× *g*, 10 min, RT) and acidified to pH 2 using 6 M HCl. The FA was extracted three times with 3 mL ethyl acetate. The combined extractions were dried under nitrogen followed by dissolution in 500 μL of H_2_O/MeOH solution at a 1:1 ratio. Twenty microliters of sample was injected for HPLC (Waters Alliance 2695, Waters Corporation, Milford, CT, USA) with a Capcell Pak C18 UG120 column (250 × 1.5 mm, 5 μm). The solvent system was composed of solvent A—0.1% (*v/v*) trifluoracetic acid (TFA) in water and solvent B—0.1% (*v/v*) TFA in methanol. The flow rate was 0.8 mL/min, and detection was performed on the absorbance at 320 nm. Elution was started isocratically at 35% solvent B, and then a linear gradient from 35% to 95% solvent B for 25 min was applied.

### 2.5. Two-Dimensional Gel Electrophoresis (2-DE) of WWDs

Two DE WWD protein analyses were performed by the methods from Kim et al. [30]. WWD proteins were extracted with extraction buffer (7 M urea, 2 M thiourea, 4% (*w/v*) CHAPS, 1% (*w/v*) DTTT, 2% pharmalyte, and 1 mM benzamidine). Protein was extracted for 1 h with vortexing followed by centrifugation (13,400× *g*, 1 h, 25 °C). The upper layer was used for two-dimensional electric motion, and the protein concentration was determined by the Bradford method [31]. The solution with 7 M urea, 2 M thiourea containing 2% CHAPS, 1% DTT and 1% pharmalyte was used for equilibrating IPG dry strips (4–10 NL IPG, 24 cm, Genomine, Pohang, Korea) for 12–16 h, and 200 μg of each sample was loaded. Isoelectric focusing (IEF) was performed at 20 °C by a Multiphor II electrophoresis unit and EPS 3500 XL power supply (Amersham Biosciences, Piscataway, NJ, USA) following the manufacturer’s instructions. For IEF, the voltage was linearly increased from 150 to 3500 V over 3 h for sample entry, followed by a constant 3500 V with focusing complete after 96 kVh. Before the second dimension, strips were incubated for 10 min in equilibration buffer (50 mM Tris-Cl, pH 6.8 containing 6 M urea, 2% SDS, and 30% glycerol), first with 1% DTT and then with 2.5% iodoacetamide. Equilibrated strips were placed onto SDS-PAGE gels (20 × 24 cm, 10–16%). SDS-PAGE was performed using a Hoefer DALT 2D system (Amersham Biosciences, Little Carlfont, UK) according to the manufacturer’s instructions. The 2D gels were run at 20 °C and 1700 Vh. Then, 2D gels were silver stained as described by Oakley et al. [32], but the fixation and sensitization step with glutaraldehyde was omitted. Quantitative digitised image analysis was carried out using PDQuest (version 7.0, BioRad, Hercules, CA, USA). The quantity of each spot was normalised by the total valid spot intensity. Protein spots were selected based on the significant expression variation deviated over twofold in its expression level compared with the non-refrigerated storage control.

### 2.6. Resistance to Extension and Extensibility of WWDs

Dough resistance to extension and extensibility were measured using a texture analyser (TA-HD plus, Stable Micro System Ltd., Haslemere, UK) by the method from Barros et al. [33]. The resistance to extension (N) and extensibility of the WWD strip (170 mm × 4 mm × 2.2 mm, length × width × thickness) were measured using a probe (Kieffer dough and gluten extensibility rig, Haslemere, UK) in tension mode at a pretest speed of 2.0 mm/s, test speed of 3.3 mm/s, posttest speed of 10.0 mm/s, and distance of 50 mm. Five replications were conducted. 

### 2.7. Dynamic Rheological Measurements of WWDs

Dynamic rheological properties were measured using a MCR 102 rheometer (Anton Paar, Graz, Austria) according to the method from Kaur et al. [34]. The plate system consisted of two plates with a 25 mm diameter with a 2 mm gap. The dough sample after resting for 1 h was placed between the plates, and the excess sample was removed. The sample surface was lightly coated with oil to prevent drying. After the sample rested for 10 min at 25 °C, the linear viscoelastic region (LVR) in all samples was determined by a frequency sweep test (1 Hz at 25 °C). Dough sample G′ (elastic modulus), G′ (viscous modulus), and tan δ (loss factor) were determined.

### 2.8. Cooking Properties of WWN

Each 30 g of noodles was cooked for 15 min in 500 mL of distilled water. After cooking, the noodles were removed from the water and drained for 15 s using noodle baskets. Cooking parameters were calculated by the method from Tudorica et al. [35]. The remaining water was used for measuring cooking loss (%). (Cooking loss (%) = (weight of remaining water which was dried overnight at 105 °C/cooked noodle weight) × 100). The noodles in the basket were rinsed in distilled water at room temperature (20 °C), and the baskets were shaken up and down for 30 s then drained for 15 s. Next, the baskets were transferred to a sink with ice water and again shaken for 30 s. Finally, they were drained for 15 s, and the excess water was removed by placing the baskets on a towel. The final cooked noodle weights were recorded to calculate the noodle water absorption (noodle water absorption (%) = {(cooked noodle weight − weight of raw noodle weight)/raw noodle weight} × 100). Ten grams of the cooked noodles were used to measure the swelling index (swelling index = (cooked noodle weight − dried noodle weight, which were dried overnight at 105 °C)/dried noodle weight) × 100). The remaining noodles were placed on a plate and covered with plastic wrap. After resting for 10 min, the cooked noodle colour and texture profile analysis was conducted.

### 2.9. Texture Profile Analysis (TPA) of Cooked Noodles

The texture profile analysis (TPA) of cooked noodles was performed using a TA-XT plus texture analyser (Stable Micro System Ltd., Haslemere, UK) using the method described by Niu et al. [26] with some modifications. After the noodles rested for 10 min at RT, one noodle strand was cut to a size of 2.5 mm, placed on a flat metal plate, and compressed twice to 75% of the noodle thickness using a cylindrical probe (P/35, 3.5 cm diameter). The pretest speed, test speed, and posttest speed were 2, 2, and 2 mm/s, respectively. The mean value of eight replications was used.

### 2.10. Statistical Analysis

Three replications of Kieffer extensibility, dynamic rheological measurement, cooking parameters, and TPA as well as duplicate WEAX physicochemical property measurements were performed. Resulting data were represented as the mean ± standard deviation. Analysis of variance (ANOVA) was carried out using XLSTAT (2016, Addinsoft, Paris, France) to determine the differences among samples. When significant differences were detected, the Student–Newman–Keuls (SNK) multiple comparison test was performed to separate the means at *p* < 0.05. Two-way analysis of variance (ANOVA) was performed to investigate the effect of enzyme concentrations and refrigerated storage time.

## 3. Results and Discussion

### 3.1. Physicochemical Properties of Water Extractable Arabinoxylan in WWDs

**Figure 1:**
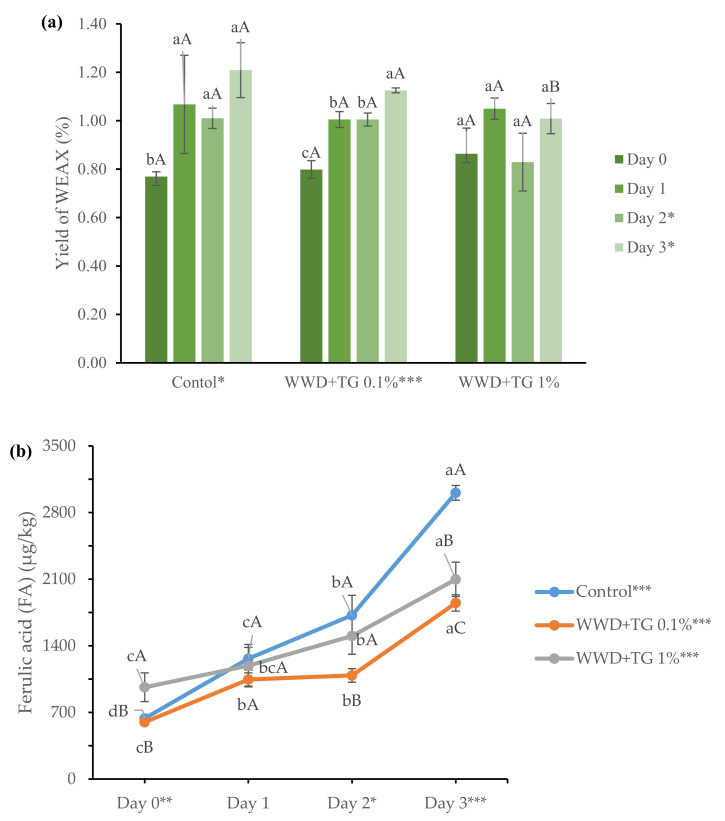
(**a**) The yield of water extractable arabinoxylan (WEAX) from whole wheat dough (WWD) and (**b**) the content of ferulic acid (FA) in WEAX from WWD with various concentrations (0, 0.1 and 1%) of transglutaminase (TG) and storage times at low temperature. Values with same lower cases are not significantly different among refrigerated storages within WWD sample, while same upper cases are not significantly different among WWD samples within a storage time. *, **, and *** are significantly different at *p* < 0.05, *p* < 0.01, and *p* < 0.001, respectively.

The WEAX yield (%) from WWDs is represented in Figure 1a. The result of two-way ANOVA for the effect of enzyme concentration and refrigerated storage time on the properties of WEAX is in Supplementary Table S1. According to Table S1, storage at low temperatures and TG concentrations influenced the WEAX yield (%). The WEAX yield (%) from WWD with 0.1% TG or the control was affected by storage time, while that with 1% TG was not affected by storage time. The range of WEAX yields was 0.75–1.32%, which agreed with WEAX yields (0.6%) from Hartmann et al. [36] who analysed WEAX from wheat flour dough. The WEAX yield (%) in WWD with 0.1% TG or that in the control increased with storage time. Our results supported those of Simsek et al. [21], who reported increased water extractable solids with longer refrigerated storage. This might imply that water migration or native enzyme action during refrigerated storage induced AX decomposition, increasing WEAX yield. However, a significant difference in WEAX content was not observed in refrigerated WWD with 1% TG. When comparing the effect of TG concentration (0, 0.1, and 1%) on WEAX yield (%) at each storage time, the difference among samples was found only on storage day 3. The WEAX yield (%) in WWD with 1% TG was lower than that with 0.1% TG or that without TG at storage day 3. This suggests that TG inhibits AX breakdown in WWD.

The content of ferulic acid (FA) in WEAX from WWD depended on TG concentration, ranging from 636 to 3007 μg/kg, and storage time at low temperature, as shown in Figure 1b. The FA content in this study was different from that by Hartmann et al. [36], who reported 403 μg/kg of FA content in WEAX from refined flour dough. The difference in FA content could be explained by the component difference between whole wheat flour and refined wheat flour. Compared to refined wheat flour, more fibres in whole wheat flour might contribute to the higher FA in WEAX. Overall, WEAX FA increased with time when stored at low temperatures. However, there was no difference in FA content among WEAX from WWD with various TG concentrations on the first day of refrigerated storage. WEAX FA is relevant to the association between soluble AX and gluten through its double bond [23]. Since endoxylanase or water migration weakens the association during refrigerated storage, FA could also be released from the network, increasing the WEAX FA content. However, when TG was incorporated into the WWD, the FA release was diminished after 2 days of refrigeration. This might be explained by the TG function, which catalyses the isopeptide bond within the gluten network, preventing endoxylanase action and maintaining FA in the WEAX-gluten network structure. The TG effects on WEAX FA were distinct with prolonged storage time at low temperature. Our results indicated that TG stabilised WWD with decreased FA difference during refrigeration and TG incorporation.

The WWD WEAX Mw is presented in Table 1. WEAX Mw (mean ± standard deviation) ranges on day 0 and after 3 days of refrigerated storage were 105,406 ± 2383 to 109,692 ± 1179 and 93,209 ± 1728 to 96,971 ± 834, respectively, which implied WWD WEAX decomposition during refrigeration. A significant difference in Mw among samples was not found on day 0. However, after 3 days of refrigerated storage, a significantly higher Mw was found in WEAX from WWD with TG than in the control. These results suggested that TG inhibited WWD WEAX decomposition during refrigeration.

### 3.2. Apparent Viscosity (AV) of WEAX Solutions

**Figure 2:**
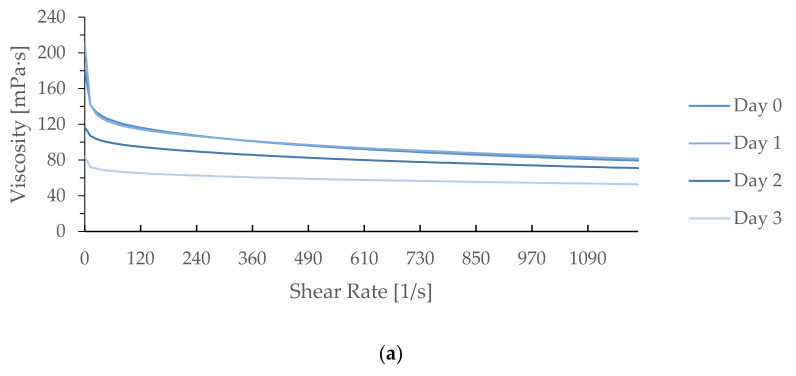

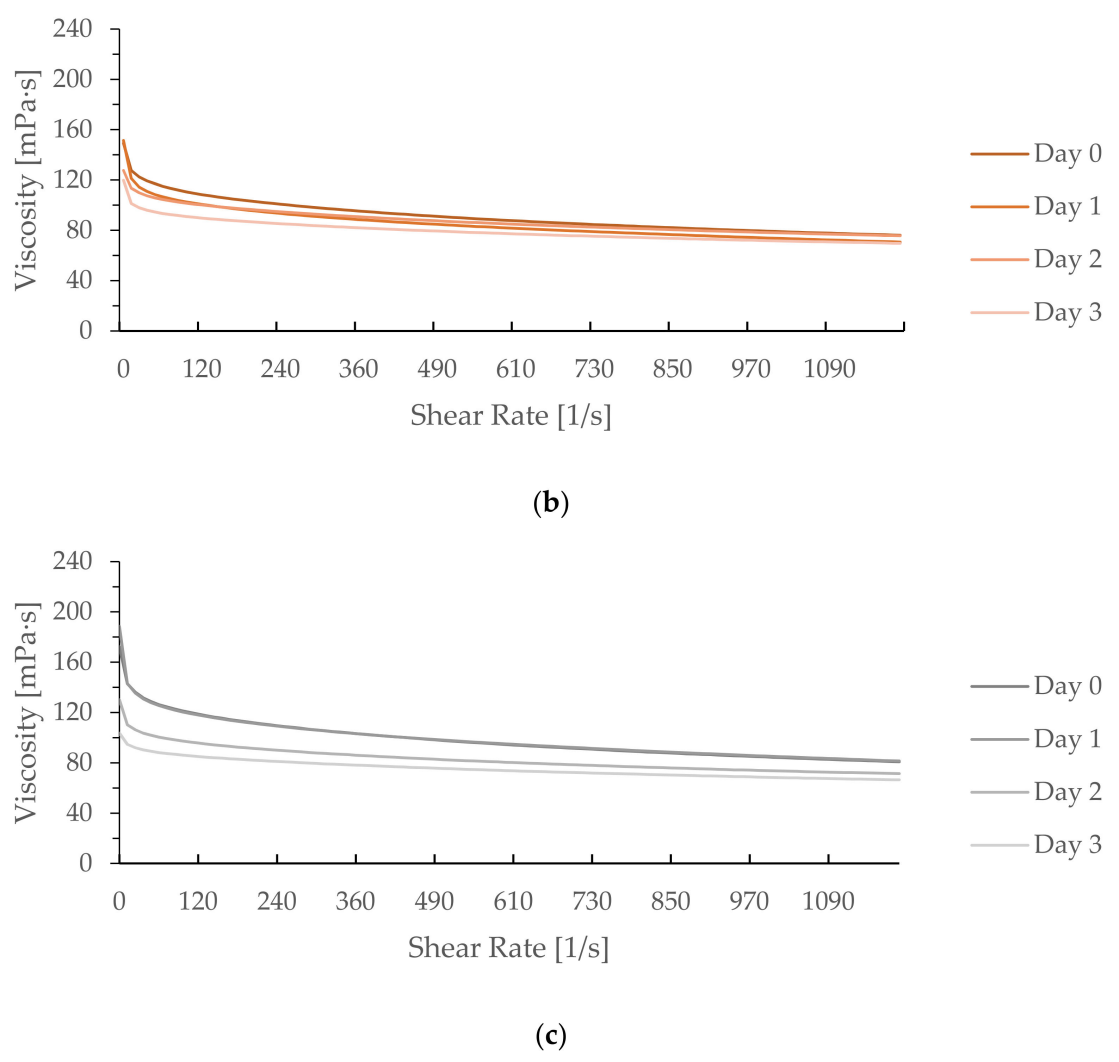
Effect of shear rate on apparent viscosity of aqueous solutions of WEAX (4% *w/v*) at 25 °C. (**a**) WWD (control), (**b**) WWD + TG0.1%, (**c**) WWD + TG1%.

Figure 2 shows the AV of the WEAX solution (4%, *w/v*) depending on the shear rate. The AV of the WEAX solution (4%, *w/v*) in WWD showed Newtonian flow characteristics at a high shear rate (600–1200/s) after 3 days of refrigerated storage. Regardless of storage time, shear thinning behavior was observed as the shear rate was increased. At the state of rest, the un-crosslinked molecule polysaccharide solution contracts to shape balls, causing high viscosity. However, when shear is applied to a polymer solution, the ball unfolds and becomes an ellipsoid [37]. The changed structure penetrates and disentangles the molecules, reducing the flow resistance and viscosities [38]. Therefore, the viscosities of the overall samples decreased as the shear rate increased. These results confirmed reports from Simsek et al. [21], which showed a similar WEAX viscosity pattern for dough refrigerated for 34 days. However, when TG was incorporated into WWD, the overall AV difference in the WEAX solution was reduced during refrigerated storage. AV of WEAX from WWD was lower than that of WEAX from WWD from TG 0.1% or TG 1% at each shear rate. This result represents a similar tendency with physicochemical properties of WEAX in WWD which demonstrates decomposition of WEAX of control occurred more than WWD incorporated with TG. In other words, the lower viscosity of control implies decomposed polysaccharides including lower resistance to shear rate. The AV difference depending on storage time for WWD with 0.1% TG was smaller than that for WWD with 1% TG. Further research is needed to explain why the AV difference depending on storage time for WWDs with 0.1% TG was smaller than that for WWDs with 1% TG.

### 3.3. Two-Dimensional Electrophoresis of WWD Proteins

**Figure 3:**
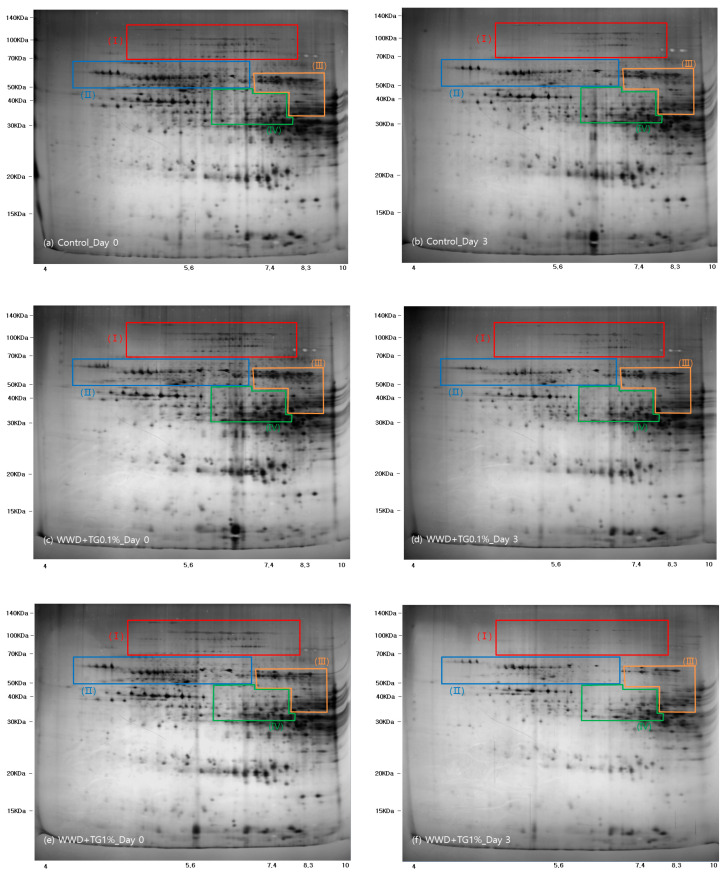
Images of 2-dimensioanl gel electrophoresis from WWDs with various concentrations (0, 0.1 and 1%) of TG and refrigerated storage time (0 3, days). The squares of (**Ⅰ**), (**Ⅱ**), (**Ⅲ**) and (**Ⅳ**) indicate high molecular weight-glutenin subunits, ω-gliadin, low molecular weight-glutenin subunits, and α, β/γ-gliadins, respectively. (**a**) Control Day 0, (**b**) WWD + TG0.1%_Day 0, (**c**) WWD + TG1%_Day 0, (**d**) Control Day 3, (**e**) WWD + TG0.1%_Day 3, (**f**) WWD + TG1%_Day 3.

Protein extractions of each of the groups with 0 and 3 day WWDs were separated by 2-DE, as shown in Figure 3. The Mw and isoelectric point (pI) were in the ranges of 10–140 kDa and 4–10 pI, respectively. High molecular weight-glutenin subunits (HMW-GS), ω-gliadin, low molecular weight-glutenin subunits (LMW-GS), and α, β/γ-gliadins separation, was performed according to Vensel et al. [39]. Glutenin 2 DE images showed that the overall intensity was decreased after three days of refrigeration. Steffolani et al. [40] reported that the TG function, changes the gluten structure and modifies SDS solubility. Their SDS-PAGE band indicated that a higher TG concentration (0.5%) had a lower intensity than a lower TG concentration (0.01%). Likewise, in this research, the 2-DE image intensity from the TG treatment group was also decreased compared to that of the control group. Figure 3c,d show differences between (III) LMW-GS and (IV) gliadin. These differences imply that the low Mw gliadin or glutenin, which were gluten components, were reduced by the TG aggregation function after three days of refrigeration. Figure 3c,d also show a similar fraction II intensity, which was a sulfur-poor polymer. Since TG contributes to disulfide bond formation related to sulfur groups, this result implies that TG affected the gluten sulfur-poor fractions less. However, the modification of SDS solubility by TG resulted in variation of 2-DE expressions. Therefore, further research on the TG effects on WWD is needed to compare the protein quantity for each fraction, namely glutenin and gliadin.

### 3.4. Resistance to Extension and Extensibility of WWD

**Figure 4:**
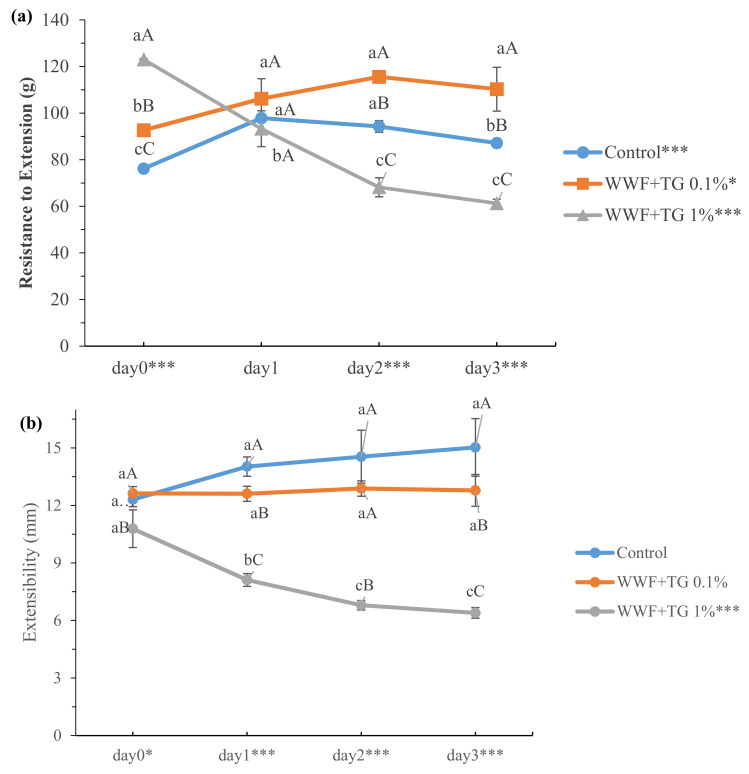
(**a**) Resistance to extension and (**b**) extensibility of WWDs with various TG concentration (0, 0.1, and 1%) and refrigerated storage time (0, 1, 2, and 3 days). Values with same lower case are not significantly different among refrigerated storage within the WWD sample, while the same upper case instances are not significantly different among WWD samples within a storage time. *, *** Significantly differ at *p* < 0.05 and *p* < 0.001, respectively.

Figure 4 illustrates the WWD resistance to extension and extensibility, which ranged from 61.28 to 123.02 g and 6.39 to 15.02 mm, respectively. Significant differences (*p* < 0.001) in WWD resistance to extension and extensibility were observed by refrigerated storage time (0, 1, 2 and 3 days) and TG concentration (0, 0.1 and 1%), as shown in Table S1. However, the tendency induced by the refrigerated storage time differed among the samples. The resistance to extension increased with refrigerated storage time in the control or 0.1% TG WWD, while it decreased with refrigerated storage time in the 1% TG WWD. WWD with TG 1% was relatively stiff, which is lacking in flexibility resulting in decreased extensibility. Also, breakage of dough occurred easily when measuring the resistance to extension, resulting in decreased resistance to extension during refrigerated storage in this study. Zhang et al. [16] reported that the resistance to extension and extensibility of refrigerated refined flour doughs were in the range of 40–90 N and 30–80 mm, respectively, which were higher than those in this study. These differences could be explained by the difference between refined flour and whole wheat flour. However, the increased resistance to extension of control and 0.1% TG on WWD were similar to the results from Zhang et al. [16]. In general, there is a negative correlation for refined flour dough between resistance to extension and extensibility [41]. Nevertheless, both the resistance to extension and extensibility of 1% TG WWD decreased with refrigerated storage time. These results might be explained by the interaction between gluten and excessive TG reaction and wheat bran interruption, increasing dough stiffness. Moreover, the extensibility of the control or 0.1% TG WWD was not changed by refrigerated storage time. Overall results of this study indicated that WWD with TG 0.1% was relatively stable in resistance to extension and extensibility during refrigerated storage compared to control. Our results suggested that adding 1% TG had a negative influence on the gluten extensibility and excessive aggregation. Therefore, a TG concentration less than 1% should be used for proper WWD extensibility during refrigeration.

### 3.5. Rheological Measurements of WWDs

**Figure 5:**
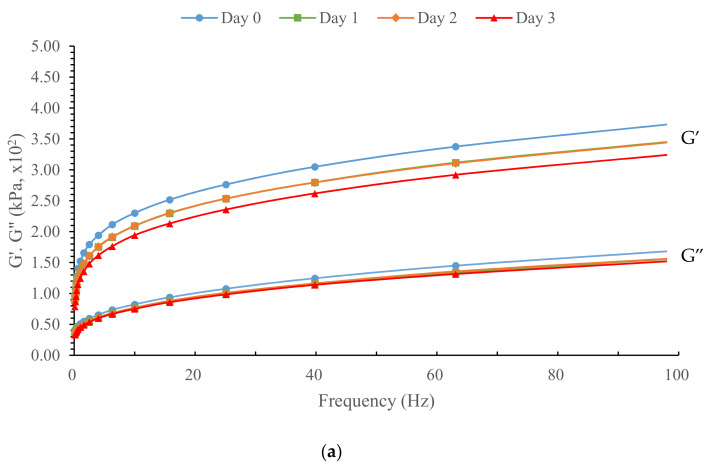

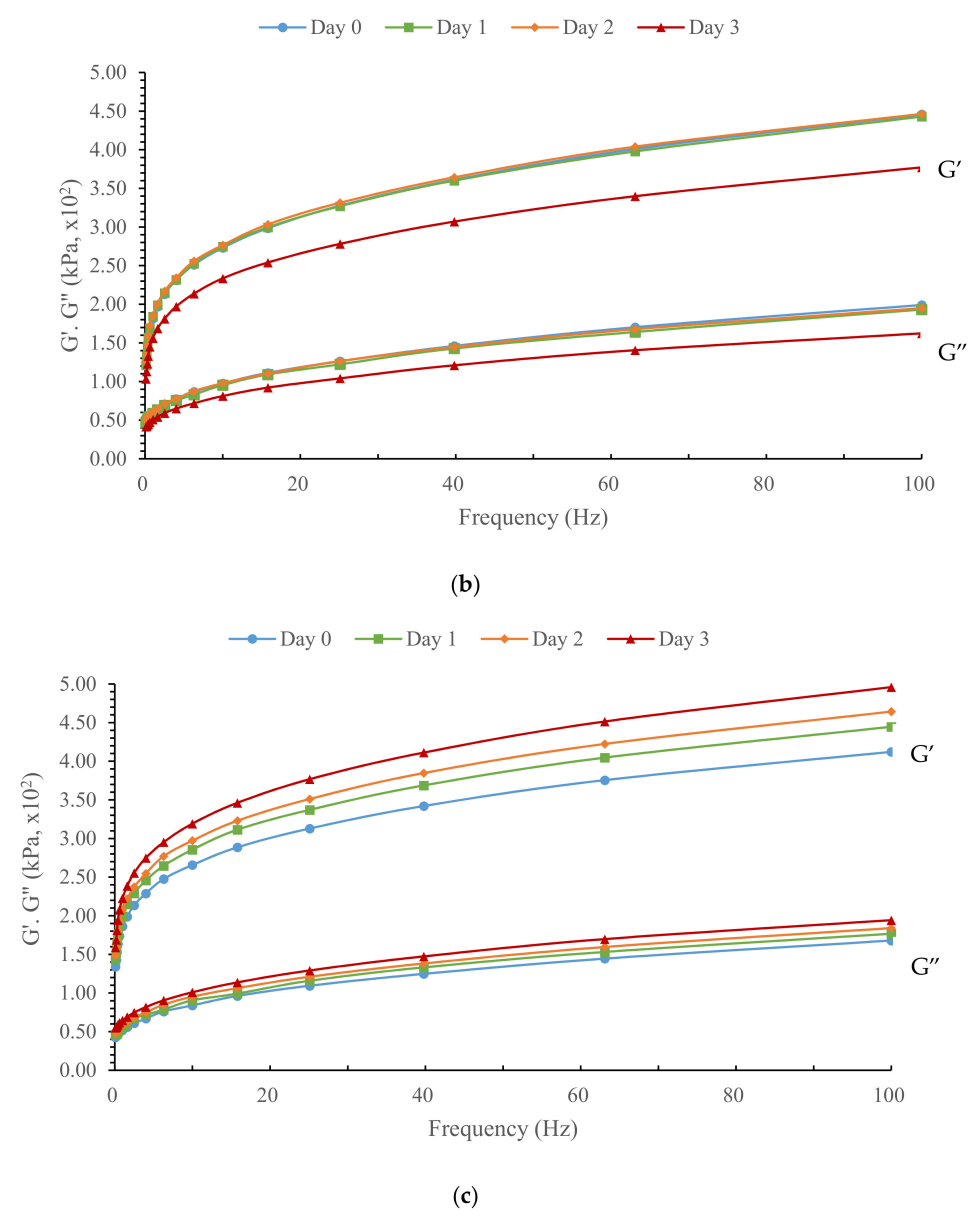
Storage (G′) and Loss (G″) modulus of WWD during refrigerated storage (0, 1, 2, and 3 days) as a function of frequency. (**a**) Control, (**b**) WWD + TG0.1%, (**c**) WWD + TG1%.

Figure 5 shows the WWD storage/elastic (G′) and loss/viscous (G″) moduli determined by the frequency sweep test depending on TG concentration (0, 0.1 and 1%) and refrigerated storage time (0, 1, 2 and 3 days). All samples had higher G′ than G″ during all storage periods, indicating more elastic than viscous properties. In addition, both G′ and G″ increased with frequency. In particular, G′ was affected by TG concentration and refrigerated time. During refrigerated storage, G′ of WWD with TG 1% was increased, while that of WWD was decreased. Whitney et al. [17] reported an identical result when investigating refrigerated dough with different levels of GOX. However, TG induced a disparate dynamic modulus pattern during refrigerated storage compared to GOX. WWD with 0.1% TG maintained its elastic modulus (G′) for two days of refrigerated storage, while 1% TG WWD showed an increased viscoelastic modulus during refrigeration. Considering that gluten structure degradation reduces elastic modulus (G′), the reason for the increased G′ could be explained by TG which formulates disulfide bonds within gluten. The 0.1% TG WWD elastic modulus was not reduced after two days of refrigerated storage. Regarding the high correlation (*r* = −0.809) between the WWD elastic modulus at 1 rad/s and extensibility in this study, TG, which prevents an elastic modulus decrease and gluten degradation during refrigeration, might prevent a dough strength decrease. However, to validate the TG function, which inhibits the gluten degradation induced by refrigeration storage, further study on the interaction between TG behavior and gluten is needed.

The average loss factor (tan δ) at all frequency points, equal to G″/G′, was in the range of 0.31–0.40 in Supplementary Table S2. During refrigerated storage, the loss factor (tan δ) was different (*p* < 0.001) depending on the WWD TG concentration. The tan δ ranges for the control, 0.1% TG, and 1% TG groups were as follows: 0.37–0.40, 0.36, and 0.31–0.32, respectively. A decreasing trend in loss factor was shown by TG. These study results imply that TG could influence gluten degradation, affecting dough strength during refrigerated storage. 

### 3.6. Cooking Properties of Noodles

Table 2 shows the WWN cooking parameters. The WWN water absorption (%) during cooking was different depending on the TG concentration and refrigeration time. The water absorption (%) was in the range of 76.35–98.31%. Regardless of the WWN TG concentration, the WWN water absorption (%) decreased with refrigerated storage time. The water absorption result contradicted that of WEAX in this study. WEAX, which has a strong water holding capacity, increased with refrigerated storage time. Even though the WEAX quantity increased, the WEAX function might have been modified during refrigerated storage, changing the water holding capacity. At each storage time, WWN with TG had lower water absorption than the control. Therefore, the WWN water absorption during refrigeration might have been affected more by the WWD gluten structure than by the WEAX amount. These results confirmed the report from Niu et al. [7], who showed reduced cooking yield (=mass ratio before and after cooking of noodles) with increased WWN TG concentrations. WWN with TG had significantly higher cooking loss and a lower swelling index than the control. This implied that TG in WWD might indirectly affect raw noodle component release more, decreasing the noodle swelling and water holding capacity. This could be partly explained by the TG function in the WWN increased compact and firm structure as TG formed isopeptide bonds within the gluten structure. However, considering that TG is involved in producing a compact gluten structure, further research on how TG affects noodle components is needed.

### 3.7. Texture Profile Analysis (TPA) of Cooked WWNs

Table 3 shows the cooked WWN TPA. Noodle hardness, adhesiveness, springiness, cohesiveness, chewiness, and resilience were determined. During refrigerated storage, a significant difference in hardness was detected only in the control noodles. A significant difference among refrigerated storage times was not found in the hardness and chewiness of WWN with TG. A significant decrease in hardness and chewiness was found in the control during refrigerated storage, while hardness and chewiness of WWN with TG 0.1% or 1% were not changed. Control and WWN with TG 0.1% were similar in hardness at day 0. However, after 2 days of refrigerated storage, WWN with TG 0.1% was similar to WWN with TG 1% in hardness and chewiness, which was higher than the control. Bread hardness from refrigerated dough increased during refrigeration due to moisture loss [42]. When comparing various TG levels (0, 0.1, 0.2, 0.4, 0.8 and 1.6%), bread hardness was increased at TG 0.8%, while TG 0.4% was suggested as a proper level for dough and bread quality [43]. However, in this study, WWN hardness from the control group decreased during refrigerated storage. The control WWN hardness was lower after two to three days of refrigeration than on day 0. Similar trends were shown in the WWN water absorption (%) in Table 2. The correlation coefficients among the WWD rheological properties and WWN TPA are shown in Supplementary Table S3. The low water holding capacity in noodles induced the high stress needed to attain noodle deformation [44], supporting the correlation of hardness with water absorption (r = −0.677) and swelling index (r = −0.758) in this study. This result indicated that the noodle hardness had a high correlation with the elastic modulus (r = 0.719) and loss factor (r = −0.801). In other words, the decreased hardness was influenced by the viscoelastic properties of dough, which were modified by the starch decomposition during refrigeration. Therefore, noodle hardness might be determined by the interaction between water holding behaviour and structural component aggregation in dough. Starch crystallization induces a low swelling index in noodles [45], while in this study, the TG reaction with gluten decreased water absorption and the swelling index. In addition, after two and three days of refrigeration, the WWN hardness was not reduced and was higher than that of the control. Consequently, because the WWD properties modified during refrigeration might have been inhibited by TG action on gluten, TG preserved the WWN quality during refrigeration. However, during refrigerated storage, the WWN with 0.1% TG had a significant difference in springiness, and 1% TG showed a difference in adhesiveness and resilience. While no difference was found in adhesiveness of WWN with TG 0.1% depending on refrigerated storage, adhesiveness was decreased with refrigerated storage in the control and WWN with TG 1%. In general, the control was more adhesive than WWN with TG 1% regardless of storage time. However, adhesiveness of WWN with TG 0.1% tended to be constant during refrigerated storage. No difference was found in the springiness of the control and that with TG 1% during refrigerated storage, while a slight decrease was noted in the springiness of WWN with TG 0.1% after 1 day of refrigerated storage. Overall, our results suggest that the TG application and concentration can be adjusted depending on the target WWN textural properties.

## 4. Conclusions

TG addition influenced both the WWD physicochemical properties and its product during refrigerated storage. During refrigeration, TG played an important role in modifying the WEAX structural properties by affecting its yield and Mw and prevented FA release from the gluten-WEAX structure. The WEAX yield (%) from WWD with 0.1% TG or the control was affected by storage time, while that with 1% TG was not affected by storage time. WEAX Mw ranges on day 0 and after 3 days of refrigerated storage were 105,406 to 109,692 and 93,209 to 96,971, respectively, which implied WWD WEAX decomposition during refrigeration. However, after 3 days of refrigerated storage, a significantly higher Mw was found in WEAX from WWD with TG than in the control implying inhibition of WWD WEAX decomposition by TG during refrigeration. The content of FA in WEAX from WWD depended on TG concentration, ranging from 636 to 3007 μg/kg, and storage time at low temperature. Overall, WEAX FA increased with time when stored at low temperatures. TG influenced FA in WEAX, which is positioned on O-5 of the arabinose residue, and the WEAX AV was altered, implying the modified WEAX branching degree. During refrigeration, the viscosity of the shear rate dependence for the control group continued to decline gradually, while the difference between the overall apparent viscosities depending on storage time was reduced when TG was incorporated in the WWD. Thus, WWD property improvement might be expected by the use of TG due to inhibited WEAX decomposition during refrigeration. However, when 1% TG was applied to the WWD, both resistance to extension and extensibility simultaneously decreased during refrigerated storage. This could be explained by the stiff WWD when 1% TG was added. The elastic modulus (G′) of WWD was decreased with refrigerated storage, while that of WWD with TG 1% was increased. Considering that gluten structure degradation reduces elastic modulus (G′), the reason for the increased G′ could be explained by TG which formulates disulfide bonds within gluten. In addition, after two days of refrigerated storage, 1% TG showed significantly higher cooking loss than the control and 0.1% TG. Consequently, even if TG ameliorates the WWD gluten structure or diminishes WEAX decomposition during refrigeration, the modified properties induced by TG do not always improve product quality. Significant decrease in hardness and chewiness was found in control WWN during refrigerated storage, while hardness and chewiness of WWN with TG 0.1% or 1% were not changed. Even though WWD with TG significantly maintained the WWN hardness during refrigerated storage, the TG concentration should be adjusted according to the target WWN properties since the WWN adhesiveness or cohesiveness differs with TG concentrations. This is the first report on the TG behaviour in refrigerated WWDs. However, further research on TG reaction in low-temperature storage is needed to confirm these findings.

## Figures and Tables

**Table 1:** The molecular weight (Mw) ^1^ of WEAX from WWD with various concentrations (0, 0.1 and 1%) of TG and refrigerated storage times (0 and 3 days).

WEAX Samples	Refrigerated Storage Times	
	Day 0	Day 3 **
Control	107,067 ± 4850	93,209 ± 1728 ^b^
WWD + TG 0.1% *	105,406 ± 2328 ^A^	96,516 ± 541 ^aB^
WWD + TG 1% *	109,692 ± 1179 ^A^	96,971 ± 834 ^aB^

^1^ All values represent means of two replications ± standard deviations. Values with same lower cases are not significantly different among refrigerated storages within WWD sample, while same upper cases are not significantly different among WWD samples within a storage time. * and ** are significantly different at *p* < 0.05 and *p* < 0.01, respectively. Values without lower/upper case indicate that there were no significant differences within the WWD sample, and storage time, respectively.

**Table 2:** Cooking properties of WWN with various concentrations (0, 0.1 and 1%) of TG and refrigerated storage times (0 and 3 days).

	Cooking Loss (%)	Water Absorption (%)	Swelling Index
**Control**			
Day 0	6.84 ± 0.37	98.31 ± 1.52 aA	2.46 ± 0.04 A
Day 1	6.75 ± 0.24	94.68 ± 1.54 bA	2.41 ± 0.04 A
Day 2	7.16 ± 0.26 B	94.03 ± 1.03 bA	2.41 ± 0.02 A
Day 3	7.16 ± 0.23 B	94.11 ± 1.92 bA	2.48 ± 0.02 A
**WWD + TG0.1%**			
Day 0	6.76 ± 0.19	98.08 ± 0.11 aA	2.42 ± 0.02 aA
Day 1	6.75 ± 0.28	92.78 ± 0.90 bA	2.33 ± 0.00 bB
Day 2	6.50 ± 0.10 C	90.23 ± 0.80 cA	2.29 ± 0.02 bB
Day 3	6.83 ± 0.34 B	87.64 ± 1.85 dB	2.27 ± 0.04 bB
**WWD + TG1%**			
Day 0	6.37 ± 0.07 b	87.28 ± 1.15 aB	2.24 ± 0.02 B
Day 1	7.16 ± 0.50 a	79.29 ± 2.16 bB	2.16 ± 0.03 C
Day 2	7.90 ± 0.45 aA	76.35 ± 3.11 bB	2.17 ± 0.10 C
Day 3	7.96 ± 0.20 aA	79.75 ± 0.21 bC	2.24 ± 0.02 B

All values represent means of five replications ± standard deviations. Same lower cases are not significantly different among refrigerated storages within WWD sample, while same upper cases are not significantly different among WWD samples within a storage time (*p* < 0.05). Values without lower/upper case indicate that there were no significant differences within WWD sample, and storage time, respectively.

**Table 3:** The Texture profile analysis of cooked whole wheat noodles (WWN).

	Hardness (g)	Adhesiveness	Springiness	Cohesiveness	Chewiness	Resilience
**Control**						
Day 0	4091 ± 49 aB	−44.62 ± 3.84 bB	0.81 ± 0.04	0.53 ± 0.02	1748 ± 93 a	0.23 ± 0.01
Day 1	4224 ± 37 a	−37.20 ± 3.90 abB	0.80 ± 0.03	0.52 ± 0.00 A	1759 ± 60 a	0.22 ± 0.00
Day 2	3217 ± 634 bB	−32.08 ± 3.27 aB	0.78 ± 0.05	0.54 ± 0.03	1336 ± 239 bB	0.23 ± 0.02
Day 3	3078 ± 542 bB	−35.32 ± 6.55 abAB	0.84 ± 0.00 A	0.52 ± 0.03 A	1336 ± 151 bB	0.24 ± 0.02 A
**WWN + TG0.1%**						
Day 0	4015 ± 278 B	−40.18 ± 0.61 AB	0.81 ± 0.01 a	0.51 ± 0.00	1668 ± 117	0.23 ± 0.01
Day 1	4311 ± 221	−42.98 ± 0.79 C	0.76 ± 0.03 b	0.53 ± 0.03 A	1715 ± 113	0.22 ± 0.01
Day 2	4470 ± 150 A	−45.22 ± 0.65 C	0.77 ± 0.01 b	0.50 ± 0.03	1706 ± 77 A	0.23 ± 0.00
Day 3	4342 ± 244 A	−42.93 ± 9.76 B	0.75 ± 0.01 bB	0.51 ± 0.03 A	1668 ± 13 A	0.22 ± 0.00 AB
**WWN + TG1%**						
Day 0	4899 ± 296 A	−34.96 ± 4.28 cA	0.78 ± 0.01	0.51 ± 0.04	1734 ± 347	0.24 ± 0.00 a
Day 1	4837 ± 473	−21.85 ± 0.80 aA	0.81 ± 0.4	0.46 ± 0.01 B	1793 ±136	0.22 ± 0.01 b
Day 2	5085 ± 32 A	−24.19 ± 0.30 abA	0.77 ± 0.02	0.47 ± 0.02	1832 ± 77 A	0.21 ± 0.01 bc
Day 3	4842 ± 103 A	−27.24 ± 1.13 bA	0.77 ± 0.02 B	0.45 ± 0.00 B	1677 ± 85 A	0.20 ± 0.00 cB

All values represent means of five replications ± standard deviations. Same lower cases are not significantly different among refrigerated storages within WWN sample, while same upper cases are not significantly different among WWD samples within a storage time (*p* < 0.05). Values without lower/upper case indicate that there were no significant differences within WWN sample, and storage time, respectively.

## Data Availability

The data to support the findings of this study are included within this article.

## Supplementary Materials

The following are available online at https://www.mdpi.com/article/10.3390/foods10071675/s1, Table S1: Results of two-way analysis of variance in this study, Table S2: Loss factor (tan δ) of WWD as a function of frequency, Table S3: Correlation coefficients among rheological properties of WWD and texture profile analysis of WWN.
