# Supplementary material for: The Effects of Transglutaminase and Refrigerated Storage on the Physicochemical Properties of Whole Wheat Dough and Noodles
Source: Foods. 2021 Jul 20;10(7):1675. doi: 10.3390/foods10071675 (PMC8304961; doi:10.3390/foods10071675)
Supplement: Supplementary file 1 [file foods-10-01675-s001.zip › foods-1304701-supplementary.pdf]

Table S1. Results of two-way analysis of variance in this study

Properties		TG Treatment	Refrigerated Storage
WEAX	Ferulic acid of content (μg/kg)	***	***
	Yield of WEAX (%)	***	***
Dough	Resistance to Extension (g)	***	***
	Extensibility	***	***
Color of raw noodles	L	***	***
	a	***	***
	b	***	***
Color of cooked noodles	L	***	***
	a	**	**
	b	***	***
TPA of cooked noodles	Hardness	***	***
	Adhesiveness	***	***
	Springiness	**	**
	Cohesiveness	**	***
	Gumminess	**	**
	Chewiness	**	**
	Resilience	**	**
Cooking parameters	Cooking Loss (%)	***	***
	Water Absorption (%)	***	***
	Swelling index	***	***
Frequency sweep of dough	G' (Elastic Modulus) (kPa)	***	***
	G" (Viscous modulus) (kPa)	***	***
	Loss factor (tan δ)	***	***

Table S2. Loss factor (tan δ) of WWD as a function of frequency.

		Transglutaminase concentrations		
		Control**	WWD + TG0.1 %	WWD + TG1 %
Refrigerated Storage Times	Day 0***	0.37 ± 0.00 aA	0.36 ± 0.01 A	0.32 ± 0.01 B
	Day 1***	0.39 ± 0.00 bA	0.36 ± 0.01 B	0.31 ± 0.01 C
	Day 2***	0.38 ± 0.00 bA	0.36 ± 0.01 B	0.32 ± 0.01 C
	Day 3***	0.40 ± 0.00 cA	0.36 ± 0.01 B	0.32 ± 0.00 C

All values are average of loss factor for overall range of frequency and it is shown as means of three replications ± standard deviations. Values with same lowercase letters are not significantly different among refrigerated storage times, while same capital letters are not significantly different among TG concentrations. **, *** Significantly differ at $p < 0.01$, < 0.001, respectively.

Table S3. Correlation coefficients among rheological properties of WWD and texture profile analysis of WWN

Index1	Hardness	Adhesiveness	Springiness	Cohesiveness	Chewiness	Resilience
R to Ex	0.004	**-0.488****	-0.237	**0.336***	0.022	**0.451****
Exten	**-0.727*****	**-0.642*****	0.244	**0.725*****	**-0.432****	**0.537****
CL	0.123	**0.622*****	-0.053	**-0.404***	-0.021	**-0.459****
WA	**-0.677*****	**-0.669*****	0.304	**0.745*****	-0.304	**0.477****
SI	**-0.758*****	**-0.488****	**0.356***	**0.683*****	**-0.404***	**0.358***
G′	**0.719*****	**0.353***	-0.280	**-0.639*****	**0.462****	**-0.391****
G″	**0.463****	0.064	-0.156	**-0.464****	**0.359***	-0.283
tan δ	**-0.801*****	**-0.514****	**0.353***	**0.625*****	**-0.469****	**0.387***

[1]R to Ex = resistance to extension (g); Exten = extensibility; CL = cooking loss (%); WA = water absorption (%); SI = swelling index; G′ = elastic/storage modulus (kPa); G″ = viscous/loss modulus (kPa); tan δ = loss factor.

*, **, *** Significantly differ at $p < 0.05$, < 0.01, < 0.001, respectively.
